# A history of studies of reproductive isolation between *Drosophila pseudoobscura* and *D. persimilis*

**DOI:** 10.1080/19336934.2024.2439111

**Published:** 2024-12-20

**Authors:** Stewart Leigh, Michael G. Ritchie

**Affiliations:** Centre for Biological Diversity, School of Biology, University of St Andrews, St Andrews, UK

**Keywords:** *Drosophila pseudoobscura*, drosophila persimilis, Speciation, reproductive isolation, reinforcement

## Abstract

*Drosophila pseudoobscura* and *D. persimilis* are a sister species pair that have been used as a model for studies of reproductive isolation and speciation for almost 100 years owing to their close evolutionary history, well characterized genetic differences, and overlapping geographic distribution. There are extensive analyses of both pre- and post-zygotic isolation, including studies of courtship divergence, conspecific sperm precedence (CSP) and how reinforcement by natural selection may or may not act to strengthen isolation in sympatry. Post-zygotic analyses explore the underlying mechanics of reproductive isolation; how inversions may give rise to initial speciation events and misexpression of key genes typically found within inversion regions render hybrid offspring unfit or inviable. We aim here to present a history of studies of reproductive isolation between this species pair, looking at how the field has developed over the last century and identifying the open questions and gaps within the literature.

## Introduction and aims

Alfred Sturtevant may have been the first to publish work on *Drosophila* hybrids. Working in the famous Morgan lab, his attempt to investigate the genetics of species differences of *D. melanogaster* and *D. simulans* was ‘thwarted’ by extensive post-zygotic isolation in the form of completely sterile hybrids that prevented backcrossing [1]. While Sturtevant’s efforts have been vindicated as an incredibly important study of speciation genetics [2], it was D.E. Lancefield, in [3] that cemented *D. pseudoobscura* and *D. persimilis* as then the most widely used species pair for investigating reproductive isolation and speciation, hailing back to work which described mating difficulties between the two species (at the time thought to be two different ‘races’ of *D. obscura*) [3]. In this pair, males readily court females of the other species, but females are the more discriminating sex and will enforce pre-mating isolation by rejecting heterospecific males. When hybridization occurred, results were consistent with Haldane’s rule which states that in F_1_ hybrid offspring it is the heterogametic sex that suffers severe fitness costs [4,5]. Lancefield discovered that F_1_ males of this cross were sterile and that this was likely caused by incompatibilities between the X chromosome of one ‘race’ and the autosomes of the other. However, fertile females allowed backcrossing. Since this initial work, this species pair have become an important model for research into reproductive isolation: 1) They speciated relatively recently (0.45–1.1 mya [6]) and there is now much published data on their demographic history. 2) They are sister species and *D. pseudoobscura* has populations in both allopatry and sympatry to *D. persimilis* [7]. 3) Like many *Drosophila*, they are a well-characterized genetic toolbox, with much available data on their natural histories, genetic differences, inversions, and estimates of gene flow. For example, they have been crucial in developing our understanding of the evolution of the neo-x chromosome within the *D. pseudoobscura* subgroup [8]. 4) Also like many *Drosophila* species, they are easy to rear in a laboratory, experimentally evolve, manipulate genetically, and conduct behavioural experiments with.

**Figure 1.**
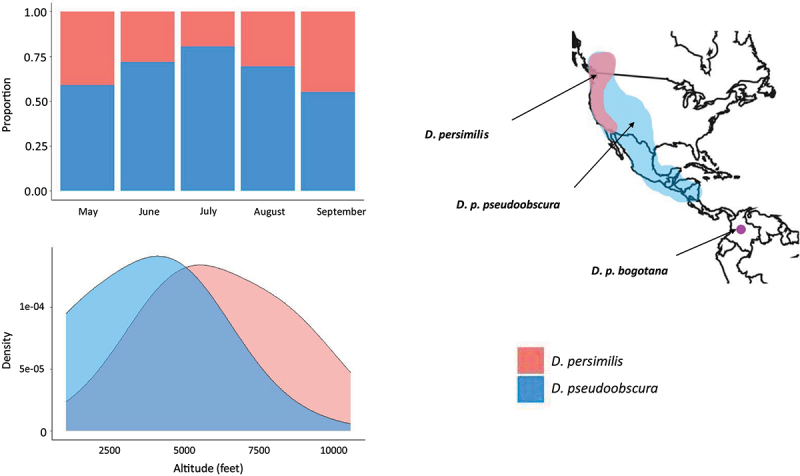
Approximate species ranges and microhabitat differences between *D. pseudoobscura* (blue) and *D. persimilis* (red). *D. persimilis* is found in the west coast of the US and British Columbia, Canada. *D. p. pseudoobscura* shares the range of *D. persimilis* but encompasses a larger range down through Mexico. *D. p. bogotana* is endemic to Bogotá, Colombia. Also shown are the relative proportion of each species captured from May to September in a sympatric range, and a density plot displaying the distribution of each species captured across an altitudinal range. Graphs created using data taken from [10,11].

Morphologically, both species appear identical except for the shape and size of the penis [9]. Both species are found in the west coast of North America; *D. pseudoobscura* consists of a main subspecies (*D. pseudoobscura pseudoobscura*) that ranges from the south of British Columbia, Canada down throughout western USA and Mexico, and another subspecies (*D. pseudoobscura bogotana*) endemic to Bogotá, Colombia. *D. persimilis* is found only in British Columbia and the west coast of the USA. *D. persimilis* populations are therefore found exclusively in sympatry with *D. p. pseudoobscura* but the wider range of *D. p. pseudoobscura* means that many allopatric populations of *D. p. pseudoobscura* exist [7] (Figure 1). Reflecting this larger range, *D. p. pseudoobscura* has a higher level of genetic polymorphism than *D. persimilis* [6,12]. Within sympatry, differences in species microhabitat have been observed with *D. p. pseudoobscura* preferring hotter dryer conditions at a lower elevation and *D. persimilis* preferring cooler wetter conditions at higher elevations [10,11,13]. There are also significant strain-specific responses to geotaxis and phototaxis (orientation and movement in response to gravity and light respectively), and dispersal behaviour, demonstrating a large amount of trait and genetic divergence between them [14–16].

**Figure 2.**
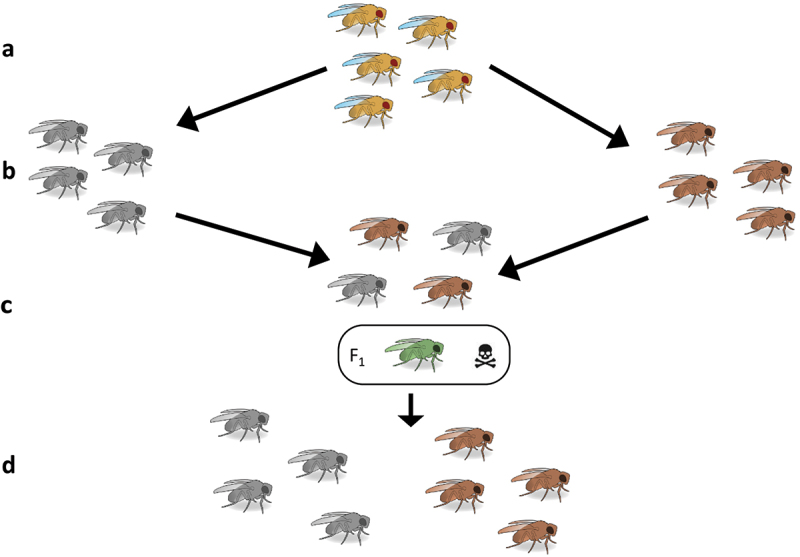
Example of how reinforcement by natural selection can maintain species barriers. a) a single interbreeding species. b) two allopatric populations of this initial species diverge into two separate species (represented by the grey and dark brown flies). c) when the two diverged species meet again in secondary sympatry, they can freely mate but F_1_ hybrid offspring are unfit or inviable (green coloured fly) which confers a high fitness cost, particularly to the parental female. d) fitness costs from hybrid mating strongly select for pre-mating barriers to increase species discrimination in females. Heterospecific matings therefore decrease in sympatry. (*Drosophila* diagrams were taken from Scidraw and created by Ann Kennedy).

Reproductive isolation describes the impediment of gene flow that evolves in diverging populations or closely related species [17]. While the ‘Biological Concept’ of species requires very strong reproductive isolation between species, most contemporary work recognize hybridization and introgression between species is relatively common. Isolation can be either pre-mating, post-mating pre-zygotic (PMPZ), or post-zygotic. Pre-mating isolation describes increased assortative mating within each distinct population or species and reduced mating between them which can be produced by divergent selection in mate preference or courtship signals (e.g [18–20]. Pre-mating isolation can also be reinforced if hybrid mating induces costs through hybrid dysgenesis which may further lead to population divergence (Figure 2) [21–23]. PMPZ regards the reproductive barriers that occur via interactions between male and female reproductive systems after mating but before fertilization has occurred. This typically comes in the form of sperm competition between males or cryptic female choice, where spermicidal, sperm ejection or mating behaviours of the female manipulate which sperm she is fertilized by, typically giving precedence to a conspecific male [24]. Finally, there is post-zygotic isolation, where genetic incompatibilities between differing species can result in hybrid offspring that suffer severe fitness and fertility costs [25]. We see evidence of all three of these in *D. pseudoobscura* and *D. persimilis* which has allowed it to endure as a model in reproductive isolation research for so long. The link between the multiple aspects of reproductive isolation and speciation is multidimensional, and many outstanding questions persist about how they are linked (nicely reviewed in [26]). For example, how much does reinforcement contribute to speciation? Or are there tipping points where weakly isolated populations become strongly isolated and speciate?

Here we do not aim to answer outstanding questions linking reproductive isolation and speciation, but to present a short history of the extensive reproductive isolation literature involving *D. pseudoobscura* and *D. persimilis* and how they have been crucial in our understanding of many of the processes involved. Our aims are threefold, first we lay out a comprehensive history of reproductive isolation research carried out using *D. pseudoobscura* and *D. persimilis*. This is split into a pre-zygotic section, with a focus on the role of reinforcement in developing and maintaining these species barriers, and a post-zygotic section that explores the genomic underpinning of reproductive isolation, including inversions and the Bateson-Dobzhansky-Muller model of incompatibility. Secondly, we focus on identifying the most prominent gaps in our knowledge and questions yet to be answered. Finally, we examine the related case study of incipient speciation between two subspecies of *D. pseudoobscura* and explore what they can tell us about burgeoning reproductive isolation.

## Pre-zygotic reproductive isolation

### Pre-mating isolation and reinforcement

Following early work to characterize differences in *D. pseudoobscura* and *D. persimilis*, Dobzhansky and collaborators studied how isolation was affected by factors such as temperature, social conditioning, and sex ratio [27,28]. They observed a higher rate of interspecific matings in laboratory strains than in natural populations, inferring increased pre-mating isolation in natural populations as a result of differences in species ecology causing the species to meet infrequently in sympatry [29]. When paired up in the laboratory, they discovered that males of both species would court and mate with F_1_ hybrid females, with *D. persimilis* males even more willing to mate with F_1_ hybrid females than conspecific females. Hybrid males are sterile but hybrid females are fertile, and this female fertility and readiness of the parental species male to mate with F_1_ hybrid females was evidence of potential genetic introgression between the two species via backcrossing [30]. Yet despite studying the phenotype and courtship of the two species, they were unable to find a physiological mechanism for reproductive isolation [31].

Considerable post-zygotic isolation exists, and not just due to male sterility (see later), so there has been much interest in these species as a potential example of reinforcement of pre-zygotic isolation. In a 1:1 male:female hybrid offspring ratio, hybrid sterility of males should render an immediate 50% fitness cost to the female but the costs associated with hybrid mating can be comprised of many different components than a simple lack of fertility in males. For example, female hybrids are fertile and can theoretically backcross into parental species (despite hybrid mating being rarely observed in wild populations) but are in worse condition, struggling to fly compared to the parental species which may impact fitness [7]. Additionally, hybrids of *D. pseudoobscura* and *D. persimilis* show a higher mutation rate than the parental species [32]. The reason for an elevated mutation rate in hybrids is unknown but Krasovec suggested two hypotheses: 1) Elevated mutation rates are a consequence of high stress and low fitness [33] which would be consistent with the observed poor condition of F_1_ hybrids. 2) Alternatively, there is evidence that mutation rates are elevated in areas of high heterozygosity [34] and being hybrid could be mutagenic due to regulatory abnormalities. Costs of hybrid mating are also likely to be multi-generational; F_2_ progeny from back crossing may be even less fertile than their F_1_ mother, possibly due to further complications from the Bateson-Muller-Dobzhansky model (see below) or through recombination in successive generations further breaking up adaptive gene complexes [35], and potentially placing estimates of the total life fitness of hybrid mating as low as 5% as that of conspecific mating [7].

Early experimental evolution work had shown that reproductive isolation quickly increased when *D. pseudoobscura* and *D. persimilis* were placed in sympatric mating cages [36] and that both stronger and weaker sexual isolation could be artificially selected for [37]. However, strictly, these studies were not demonstrating reinforcement *per se*, as they involved killing the hybrids after each generation, preventing any gene flow and ultimately simulating a scenario where speciation by post-zygotic isolation was already complete [22,38]. Gene flow is necessary in studies of reinforcement because this is the main barrier to reinforcement. Reinforcement acts to increase the frequency of alleles involved in strict species recognition, gene flow tends to homogenize genetic differences between these two speciating populations [39]. In light of this, convincing examples of reinforcement with gene flow were seen as rare in the 1970s to the 90s [22] but the potential for backcrossing between *D. pseudoobscura* and *D. persimilis* made this system particularly pertinent for investigating reinforcement around this time.

Noor’s seminal paper ‘Speciation Driven by Natural Selection in *Drosophila*’ countered some of the limitations of previous studies to demonstrate reinforcement in natural populations of *D. pseudoobscura* and *D. persimilis*. Typically in mating tests males of both species will court indiscriminately, therefore it is female choice that contributes to any reinforcement between populations [40,41]. The study involved single pair matings of individuals from stocks that were established from natural sympatric populations of both species and from allopatric populations of *D. pseudoobscura*. Experiments were carried out no more than one and a half years from the collection of the original flies, therefore the individuals tested had stemmed from a mating environment where hybrids survive and gene flow could occur. Females from sympatric populations mated with heterospecific males significantly less often than females from allopatric populations [42]. Furthermore, females from smaller populations of *D. persimilis* were less likely to mate with heterospecifics because being from rarer populations, they are more likely to encounter a heterospecific male. Under the reinforcement model this would be expected, as females that are more likely to encounter a heterospecific male are predicted to more strongly select for mating barriers to avoid costly hybrid mating.

Prospective issues have been raised in the methodology that may have resulted in factors other than reinforcement influencing the results, for example, sympatric populations are found in the west coast of the USA, whereas allopatric *D. pseudoobscura* populations were found inland further east. Therefore, differences in mating rates may have been influenced by adaptations to different environmental clines between populations [43]. Furthermore, these results are puzzling in the face of work by Stephen Schaeffer et al. who found gene flow between allopatric and sympatric natural populations of *D. pseudoobscura* across the whole genome except for the highly polymorphic chromosome 3 which shows differences in inversion frequencies across the species range including between sympatry and allopatry [44,45]. It seems likely that any reinforcement has not had a large difference on the extensive colinear genome and may be confined to the inversion, though more detailed identification of genes contributing to reinforcement, along with detailed studies of genomic divergence, are needed. Despite these caveats, it remains an important and likely study of reinforcement between the two species.

Since Noor’s paper, other studies of pre-mating isolation between this pair have been more mixed. Lorch and Servedio supported pre-mating reinforcement by finding evidence of higher reproductive isolation between sympatric than allopatric populations. They also found asymmetry in the mating, with *D. pseudoobscura* females being less receptive to heterospecific males than *D. persimilis* females in sympatry [46]. Asymmetry is an indication of reinforcement as differences in range and population size may cause females of one species to encounter males of the other species, and thus experience the costly effects of hybrid mating, more frequently than the reciprocal. The results from these studies were not without criticism which led to some debate regarding the philosophy of design in mate choice experiments and how naturalistic and therefore valid any results about reinforcement in lab studies can be. Anderson and Kim criticized Noor’s study as being unnaturalistic due to the ‘no choice’ design and failed to find evidence of reinforcement in their own multi-choice experimental setup of sympatric and allopatric populations [47]. Unfortunately, their analyses pooled heterospecific matings of both species’ females against conspecific matings to calculate reinforcement. This was criticized as problematic as a) allopatric populations of *D. persimilis* do not exist and b) *D. persimilis* females are naturally less discriminating in heterospecific mating than *D. pseudoobscura* females. Therefore, heterospecific mating rates of sympatric *D. persimilis* were pooled with allopatric *D. pseudoobscura*, obscuring the effect of sympatry vs. allopatry and artificially increasing the mating index of allopatric *D. pseudoobscura* [48].

Noor and Ortíz-Barrientos reanalysed this data, separating *D. persimilis* from the calculation to look at differences between allopatric and sympatric *D. pseudoobscura* heterospecific mating specifically. From this they concluded that Anderson and Kim’s dataset did in fact show evidence for reinforcement. Anderson and Kim later refuted this reassessment of their data, arguing that all heterospecific matings were important to consider in a test of sexual isolation to create an overall picture of sexual isolation between the two species, not just one that is focused on *D. pseudoobscura*. Therefore they claimed that their original index was more realistic and as such they defended their conclusion of not finding evidence of pre-mating reinforcement between *D. pseudoobscura* and *D. persimilis* [49].

There has been little research into reinforcement in this species pair in the last couple of decades, but modern sequencing technologies allow new approaches to tackle these questions. For example, we would expect to see greater regions of divergence at loci undergoing reinforcement selection between sympatric and allopatric populations of *D. pseudoobscura* [50]. These kinds of reinforcement signatures using modern sequencing data have been instrumental in identifying regions of reinforcement across many taxa such as monkeys, mice, and frogs [51–53] and would likely help us shed light on these discrepancies in the literature. Also, new analytical methods to identify the extent and timing of gene flow between species could give new insights into this system [54].

### Mechanics of pre-mating reinforcement

It is not clear what cues females appear to be using when they discriminate against heterospecific males and thus what traits exactly may be under reinforcing selection. *Drosophila* have complex multimodal courtship behaviours that involve wing songs, orientation, tactile signals and licking [55,56], therefore as pre-mating isolation involves the rejection by a female, mating barriers may be associated with courtship divergence in many traits [57]. Study initially found genetic links to mating barriers, localizing species specific preferences to the X and 2^nd^ chromosome [57]. In the 1960s, differences in the low and high repetition songs in *D. persimilis* and *D. pseudoobscura* were isolated to genes in the X chromosome [58]. Later a correlation between wing song differences and species-specific inversions on the 2^nd^, 3^rd^ and X chromosome was found. Different species’ females appeared to prefer different elements of the wing song with *D. persimilis* females being more receptive to the intrapulse frequency and *D. pseudoobscura* females to the interpulse interval [59]. Around half of *D. persimilis* males tested displayed a Postural Display of Courtship (PDC), which is absent in *D. pseudoobscura*. It consists of movement of the abdomen and the production of a nutritional ‘droplet’, offered to the female, which she may accept to initiate courtship. The PDC is shown to increase male copulation success when a rival male is present and as it is specific to *D. persimilis*, it may aid in species recognition for the female in choosing the correct mate. Thus, PDC may potentially be part of the shift in courtship divergence between the two species [60]. As it stands, we still know little about which specific courtship factors are under reinforcement and how they diverge between species, and which of these factors females specifically use when deciding on a mating male.

**Figure 3.**
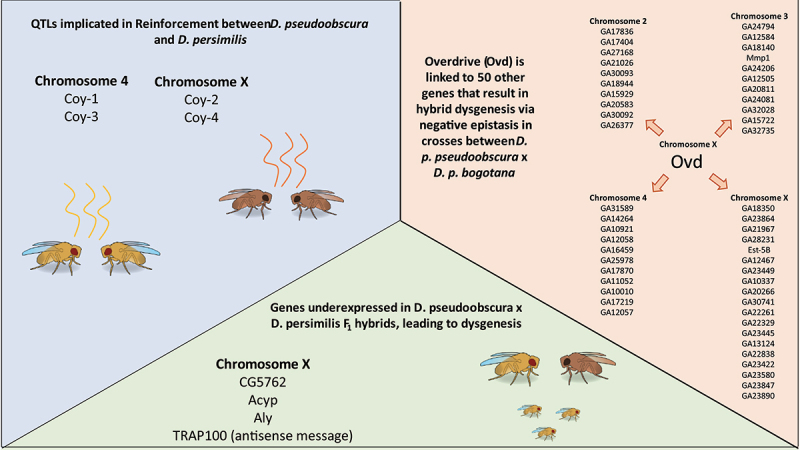
Genes that have currently been identified in studies of *D. pseudoobscura* and *D. persimilis* that are associated with reproductive isolation. In the blue box are loci that are associated with olfaction behaviours and have been implicated in reinforcement between sympatric populations of *D. pseudoobscura* and *D. persimilis* (represented here in different shades of brown) [61]. The green box shows genes implicated in post-zygotic hybrid dysgenesis [62,63]. The orange box shows the gene *Overdrive* which is part of a network of 50 other genes that cause hybrid dysgenesis in crosses of *D. p. pseudoobscura* and *D. p. bogotana* via negative epistasis [64]. In each box are the chromosomes where these genes are found. (*Drosophila* diagrams were taken from Scidraw and created by Ann Kennedy).

A dominant set of QTLs named *Coy1–4*, associated with olfaction [61], have been implicated in reinforcement behaviours (Figure 3). Introgression experiments of *D. pseudoobscura Coy-2* alleles backcrossed into *D. persimilis* from both sympatric and allopatric populations found that mating discrimination was high in *D. persimilis* but significantly higher if the copy of *Coy-2* came from a sympatric (i.e. reinforced) population of *D. pseudoobscura*. Therefore, this gene appeared to be a ‘one-allele assortative mating locus’ that had generality in reducing heterospecific mating in either species background (Ortíz-Barrientos and [63]. However, a later repeat of the 2004 study found no evidence that *Coy-1* or *Coy-2* was involved in mating discrimination within inbred laboratory strains [65]. The overall implications from these studies are that certain alleles of *Coy-1* and *Coy-2* appear to be associated in reinforcement but the failure to be captured within inbred lab strains suggest that there is high allelic variation in natural populations and other genes are likely to be contributing to reinforcement also [66]. Future studies could follow up on these results by analysing *Coy* alleles within different populations; If *Coy-1* and *Coy-2* are involved in reinforcement, then we would expect them to be at a higher frequency in sympatric populations of *D. pseudoobscura*.

Mating barriers may also include a learned component. Males of either species do not initially discriminate against the species of female that they court, but rejection by heterospecific females may result in reduced future heterospecific courtship to avoid wasting time and energy. Initial studies did not support this, showing that indiscriminately mating males have no selective pressure to learn to avoid courting heterospecific females even after being rejected [67]. However, later studies found that males from both species who were rejected by heterospecific females but later accepted by conspecific females were less likely to court heterospecific females again, with *D. persimilis* males being particularly sensitive to rejection and less likely to court heterospecific females in the future [68,69]; Kujtan and [69]. Whether the genetic component behind this learned avoidance behaviour can be reinforced is currently unknown. More generally it is interesting to consider if learning could accelerate or inhibit reinforcement. However, recent modelling in other species suggests that learned behaviours without a genetically hardwired component are a barrier to reinforcement [70].

### Post-mating pre-zygotic (PMPZ) isolation

In addition to pre-mating isolation, significant PMPZ barriers have been identified between *D. pseudoobscura* and *D. persimilis*. These were first noticed by Dobzhansky who found evidence of conspecific sperm precedence (CSP) via males transferring fewer sperm to heterospecific than conspecific females [71]. CSP can take several forms such as male allocation of transferred ejaculate, differences in sperm type or seminal fluid proteins making up the composition of the ejaculate which may aid in sperm competition. Females can also influence CSP by exerting control of the sperm used to fertilize her eggs in a process known as cryptic female choice [72]. This can be controlled through, for example, shifting mating time with the male to increase or decrease the chance of sperm transfer, ejecting the ejaculate, or spermicidal action to remove the sperm of an undesirable male [73].

PMPZ barriers in the form of gametic isolation and sperm storage have been shown to be a driving force for reinforcement between heterospecific mating pairs of several species of *Drosophila* [74–76]. Evidence of CSP reinforcement between *D. pseudoobscura* and *D. persimilis* specifically is found with sperm competition; sympatric *D. pseudoobscura* ejaculate outcompetes that of *D. persimilis*, attaining a higher ratio of paternity when mated to *D. pseudoobscura* females. Sympatric *D. pseudoobscura* ejaculate is also more competitive against *D. persimilis* than *D. pseudoobscura* from allopatric populations [77].

Members of the *obscura* group including *D. pseudoobscura* are unique in that they contain two different sperm types: a fertilizing ‘eusperm’ and a non-fertilizing ‘parasperm’ [78]. The function of parasperm is not fully understood but is associated with protection of eusperm [78], and increased sperm competition success by being a cheap way of filling the female spermathecae and flushing out rival sperm [79]. Parasperm itself is categorized into two classes in *D. pseudoobscura* (parasperm 1 and 2) with parasperm 2 hypothesized to be advantageous in sperm competition due to its increased production in response to rival males [80]. Parasperm 2 is phenotypically longer and is uniquely corkscrew shaped, phenotypes that Alpern et al. suggested may be useful in displacing sperm from rival males. The parasperm of *D. persimilis* is not characterized as thoroughly as that of *D. pseudoobscura* and we do not know whether they also produce two classes.

The female reproductive tract of *D. pseudoobscura* has certainly been shown to have a spermicidal effect [81] and such observed sperm dynamism was suggested to explain CSP if conspecific parasperm could more effectively protect conspecific eusperm than heterospecific parasperm against a species specific spermicide. However, no such basis has so far been found [82]. Peckenpaugh et al. did find that heterospecific mating was shorter with sympatric, than allopatric, populations of *D. pseudoobscura*. This is indicative of reinforcement and may drive the observed CSP effect through differences in the amount of seminal fluid proteins transferred, which in turn can affect the transport, storage, and competitiveness of sperm.

In addition to modifying the behaviour of the sperm, seminal fluid proteins transferred during mating can induce several post-mating responses in the female such as reduced receptivity to further mating, increased oogenesis, and sperm toxicity [83–85] Inhibition of remating is a well-characterized method of male manipulation during conspecific matings to ensure paternity but may reduce female fitness by stopping her from remating from a higher quality male. In conspecific matings, the deleterious effects of seminal fluid proteins transferred to females may be offset by an increase in overall fitness if the proteins are necessary for proper processing of sperm and oviposition [86], but in heterospecific matings the lack of fertile or viable offspring means that any benefit from these proteins is eliminated and harmful effects are compounded. This is therefore another obvious potential driver of PMPZ reinforcement. However, despite females of both species suffering the refractory effects of heterospecific seminal fluid proteins, reinforcement to resist this in sympatry has not been found to occur [87].

PMPZ reinforcement between *D. pseudoobscura* and *D. persimilis* remains understudied and results are inconsistent. Going forward it will be important to fully characterize sperm types for both species and identify any differences in composition or function that may be important in CSP. Modern proteomic techniques are perfectly sufficient to tackle these questions. Particularly, differences in heterospecific seminal fluid protein makeup and allocation will be able to confirm hypotheses on the underlying mechanisms of CSP and how differences in species specific sperm are processed and responded to by females.

### Costs of reinforcement

While there appears to be a role for CSP in reproductive isolation between *D. pseudoobscura* and *D. persimilis*, intraspecific sperm competition between allopatric and sympatric *D. pseudoobscura* males (i.e. sperm competition within a *D. pseudoobscura* female) results in significantly more offspring from allopatric males. Thus sympatric males becomes less competitive than allopatric males in conspecific matings [77,82]. This suggests that reinforcement is costly, conferring an advantage only when the costs of hybrid mating are sufficiently high. Such a cost to reinforcement is poorly understood, and in general, would seem to provide a substantial obstacle to ‘cascade’ reinforcement, when increased sexual isolation is thought to spread to allopatric populations [88].

Reinforced pre-mating barriers also appear to weaken in experimental secondary allopatry [89]. Heterospecific mating barriers of laboratory stocks established from sympatric populations collected in the 1950s, 1990s and 2000s were found to be decaying, with *D. pseudoobscura* females in particular becoming more receptive to heterospecific mating over time. While Myers and Frankino highlighted a potential effect of drift in inbred lab populations, they also concluded that mating discrimination alleles are likely to be costly and purged under relaxed selection. While evidence infers a cost for pre-mating reinforcement, we do not currently know what these costs may be or how costly these reinforcement genes are; therefore, we also do not know how costly hybrid mating must be before reinforcement genes become advantageous within a population.

Genes involved in post-mating interactions are predicted to evolve very quickly due to sexual selection and antagonistic coevolution [90] and this rapid evolution may also confound the spread of reinforcement genes in natural populations, especially if reinforcement is costly or selection pressure is too weak [87]. Some evidence even suggests that hybrids could be fitter than the parental strains later in life and thus sympatric reinforcement selection pressure could be far weaker than was previously thought [91]. In such a case reproductive isolation could be due to variation in factors directed by interspecies interaction or intraspecific sexual selection and not reinforcement through natural selection [89].

Sexual selection can give rise to speciation and reproductive isolation if populations diverge in traits and preferences that are being selected for. Once sufficiently diverged, secondary sexual characteristics from one species will not be attractive or even identifiable as courtship to members of another species [92–94]. This has been evidenced many times in related *Drosophila* species, with wing song, cuticular hydrocarbons (CHCs) and body size all shown to diversify to become pre-mating barriers in experimental and natural populations of *D. melanogaster* due to sexual selection [18,95–97]. Experimental evolution lines of *D. pseudoobscura* have similarly shown significant courtship divergence under different sexual selection regimes after 200 generations. High sexual selection lines evolve a faster and more vigorous wing song with a shorter interpulse interval than low sexual selection lines, and females generally co-evolve to prefer the wing song of males from their line, although this preference is asymmetric and males evolved under high sexual selection are successful in mating with females from either line [98–100]. Additionally, high sexual selection is shown to significantly affect the expression of genes associated with reproductive functions in female *D. pseudoobscura*, further demonstrating how sexual selection quickly affects the evolution of reproductive traits [101]. The crucial point is that these studies show that the forces that can lead to reproductive isolation can alternatively arise from forces other than reinforcement by natural selection.

Overall, we agree with the interpretation of Noor and Ortíz-Barrientos that pooling *D. persimilis* mating data with that of sympatric *D. pseudoobscura* will obfuscate any signal of reinforcement. We would also add that the criticism of the ‘no-choice’ experiment is also potentially flawed as females do have a choice, to mate or not to mate, and significant differences in mating rate have been found considering this. The evidence from more recent studies [46] corroborates Noor’s original findings. However, the role of reinforcement may be insufficient in itself in determining reproductive isolation. There is evidence that suggests that pre-mating and PMPZ reproductive isolation barriers can be reinforced between *D. pseudoobscura* and *D. persimilis*. However, tenuous evidence of selection pressures, inferred costs to reinforcement alleles, and confounding alternative drivers of mating barriers such as sexual selection suggest that reinforcement may be only one factor in determining the overall level of reproductive isolation. We still have an incomplete understanding of reinforcement in this species pair, and open questions remain pertaining to how costly reinforcement is and why it is, and how strong selection pressures need to be for reinforcement to evolve despite those costs. We also know very little about PMPZ reinforcement such as what the underlying mechanics of CSP in these species are and how they may be reinforced, and a full characterization of the sperm of both species, particularly in *D. persimilis*.

## Post-zygotic reproductive isolation

As previously discussed, the female fitness costs of hybrid mating can be over 50% due a combination of sterility and inviability of the hybrid offspring. The genetic basis for this hybrid dysgenesis has been studied for as long as the discovery of reproductive isolation between them, with Lancefield noticing that the sterility of the males appeared to be influenced by incompatibilities of the X and autosomes [3]. The body of work that followed to explain these incompatibilities can broadly be split into: 1) inversion differences between the two species, encompassing the low levels of gene flow and high divergence of genes within these inversions, and 2) the molecular genetic mechanisms themselves that produce unfit hybrid offspring via negative epistasis.

### Inversions

Briefly, inversions occur spontaneously due to errors in the DNA repair process [102]. They can spread through drift, through selection if they if they capture beneficial alleles (or few deleterious alleles), or they can contain a meiotic drive which pushes it via super-Mendelian inheritance as is the case with the *D. persimilis* ‘sex-ratio’ inversion (Sturtevant and [103]. The evolution of inversions is beyond the scope of this review but is summarized nicely in more focused reviews [104,105]. What is important here is how these inversions, once established within a population, can contribute to speciation and facilitate post-zygotic reproductive isolation.

*D*. *pseudoobscura* and *D. persimilis* inversions that differ between the species have now been well described. *D. persimilis* has a fixed inversion on XL that is absent from *D. pseudoobscura*. The XR of *D. persimilis* has two different gene arrangements, one inversion that is absent from *D. pseudoobscura*, and a ‘sex-ratio’ arrangement that is homosequential to the *D. pseudoobscura* ‘standard’ gene arrangement. *D. pseudoobscura* has two gene arrangements on its XR, the ‘standard’ arrangement, and a unique ‘sex-ratio’ arrangement consisting of three non-overlapping inversions. In addition to these X chromosome arrangements, *D. persimilis* has a large fixed inversion on chromosome 2 that is absent in *D. pseudoobscura* [10,12,106,107]. The 3^rd^ chromosome of both species is unique in that it displays extensive levels of polymorphism, with over 30 different structural variants identified in *D. pseudoobscura* and over 20 in *D. persimilis*. Only one structural variant on the 3^rd^, named the ‘standard’ arrangement, is common to both species [108,109].

**Figure 4.**
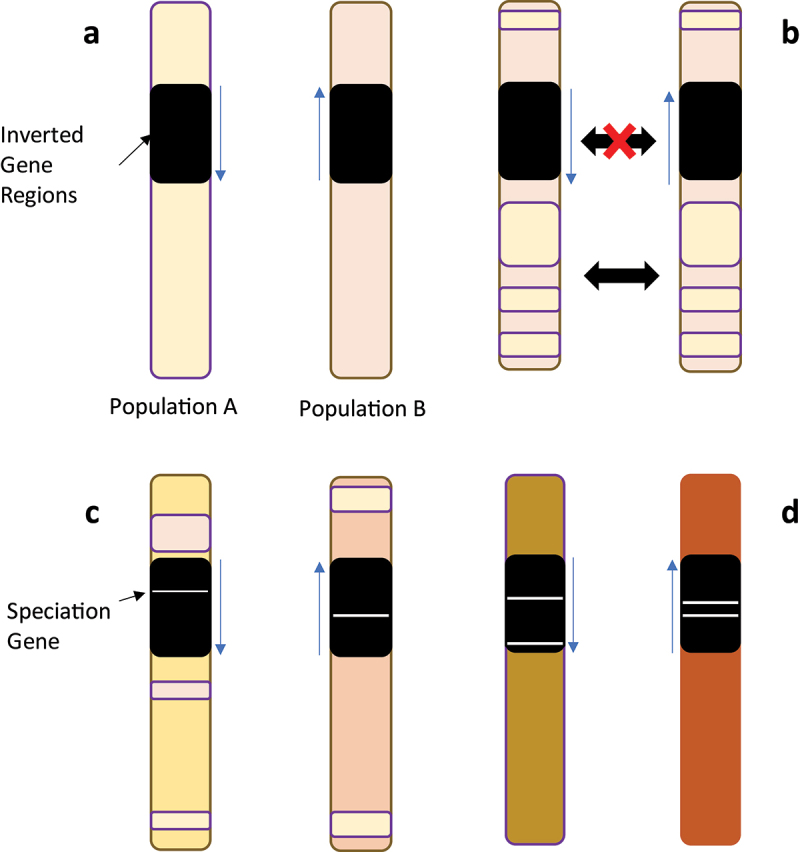
Example of how inversions can lead to speciation and reproductive isolation. A) Example of the same chromosome in two hypothetical populations of a diverging species, with an inverted gene region in black. b) populations can mate leading to gene flow in collinear regions and homogenization of the genome. However, recombination is low in inversion regions leading to suppressed gene flow in those areas. c) because gene flow is supressed, genes in these regions can vastly diverge between the two populations due to local co-adaptation, eventually causing incompatibilities when in the background of the reciprocal population. d) as these speciation genes grow, populations become sexually isolated as hybrid offspring become infertile or in poor condition. Mating becomes infrequent or eliminated, gene flow stops completely, and thus collinear regions diverge leading to two distinct species.

These species specific variants were first implicated as species barriers by Tan [57], who found that *D. persimilis* were more receptive to mating with hybrids that carried the *D. persimilis* 2^nd^ and X chromosome, i.e. the chromosomes containing *D. persimilis* specific inversions. Later evidence for inversions as a driver of reproductive isolation showed that the highest areas of genetic divergence between *D. pseudoobscura* and *D. persimilis* in the genome were located within the inversion regions due to supressed recombination between heterokaryotypes [110]. While gene flow may occur readily between collinear regions, it would do so at only a very low rate if at all within inverted regions, retaining species specific parts of the genome. Inverted regions would therefore contain the most genetically diverged genes between species, and accumulate further differences, potentially conferring hybrid sterility [111–113] (Figure 4).

The role of inversions in facilitating species barriers has been strongly supported by genomic analyses at multiple evolutionary timescales; significantly higher sequence divergence is found in inverted versus collinear regions in the face of constant interspecies gene flow from pre- to post-speciation, as well as expression divergence of genes found within those inversions [6,114]. As a result, many reproductive isolation genes have been mapped to inversion areas. Ortíz-Barrientos et al. has argued that ‘basal’ genes involved in reproductive isolation are distinct from reinforced genes as they are present in allopatric *D. pseudoobscura* populations where selection pressure for reinforced mating discrimination is not present. While reinforced genes can appear anywhere in the genome, unlikely to be weakened by rare occurrences of gene flow in already established species, basal genes map exclusively to the inversion sites. This suggests a role for basal genes in incipient speciation, evolving into initial mating barriers within inverted areas of low to no gene flow even when the two species can freely hybridize [61,115–118].

A specific example of collinear divergence is worth touching upon at this point. The faster-X effect predicts that selection on X linked genes will be stronger due to the expression of recessive alleles in the hemizygous sex and therefore experience more adaptive substitutions. Estimates of dN/dS on X-linked genes within collinear regions has shown evidence of the faster-X effect between *D. pseudoobscura* and *D. persimilis*, driving divergence between them on these regions of the X chromosome [119].

Competing hypotheses for the origin of karyotype differences between *D. pseudoobscura* and *D. persimilis* have been proposed. McGaugh and Noor [120] suggested that the fixed inversions in the XL and 2^nd^ chromosome of *D. persimilis* follows a mixed geographic model. Here the inversions originated in the allopatric population of the ancestral species that became *D. persimilis*, with locally adapted genes within inversions allowing the persistence of the inversions within the *D. persimilis* population in allopatry. Upon secondary contact with *D. pseudoobscura* post-speciation, the lack of recombination in locally adapted inversions would become a selective advantage over collinear regions, preventing adaptive gene combinations from being broken up and homogenized [121]. The proposal that these inversions arose in the *D. persimilis* lineage after the split between the species is well substantiated [117,120]. A recent study that developed high-quality long read assemblies found that 8 out 9 detected microinversions were derived in the *D. persimilis* lineage, indicating that this lineage may be prone to the development of chromosomal rearrangements [114].

Alternatively, work by Fuller et al. [122] argued that these inversions were already present in the ancestral species prior to the lineage split that would speciate into *D. persimilis*/*D. pseudoobscura*. Their model states that inversions could have begun to diverge from the equivalent collinear regions in the heterozygous state due to supressed recombination. Incomplete lineage sorting may then have resulted in different karyotypes segregating within differing populations of the ancestral species. Thus, the lack of recombination already present in the species-specific karyotypes would have been fertile ground for hybrid incompatibility genes to evolve.

It is difficult to clearly resolve these differing models. Studies which can time the origin of inversions relative to speciation events are needed and identifying the timing of inversions with respect to speciation events is not straightforward (e.g [123]. Such studies in a different system, *D. mojavensis*, have shown that major inversions show similar timings as the species [123], but it is clear that some inversions systems predate speciation in other systems. Both models advocate for low gene flow within inversions housing incompatibility genes that drive speciation. If genes inside inversions are rapidly diverging, resulting in hybrid incompatibility, and promoting reproductive isolation then introgression events across the whole genome should fall over time between *D. pseudoobscura* and *D. persimilis* as reproductive barriers are strengthened.

### *Gene flow between* D. pseudoobscura *and* D. persimilis

Under certain laboratory conditions, F_1_ hybrids can occur commonly in a population and also backcross, leading to significant levels of introgression [124,125]. Despite this, in natural populations hybrid matings are rarely seen, and introgression is expected to be rarer still. Dobzhansky considered reproductive isolation to be complete with no gene flow as hybridization appears to be extremely rare in the wild and predicted that even low levels of gene flow would have been strongly reinforced against [126]. Kulathinal and Singh argued that introgression between the two species is minimal if it even occurs at all, arguing for a model of ‘reinforcement without gene flow’ [127,128]. In their study, they found that genetic similarity between *D. pseudoobscura* and *D. persimilis* did not increase under sympatry compared to allopatry (i.e. no gene flow between hybridizing species).

This conclusion was subsequently questioned on the basis of Kulathinal and Singh’s analysis being too insensitive to pick up low levels of interspecific gene flow among high levels of intraspecific gene flow, and looking at a small subset of genes primarily located on chromosomes not homosequential between *D. pseudoobscura* and *D. persimilis* [129]. Wang et al. had previously inferred that gene flow was very variable between loci, and demonstrated limited but ongoing gene flow in the *Period* and *Alcohol dehydrogenase* (*Adh*) loci between *D. persimilis* and *D. pseudoobscura* [130,131], and Noor later showed that while gene flow would be very unlikely to occur within inverted regions due to low recombination rates and the presence of barrier loci, if there is hybridization then it should be detectable within autosomal collinear regions [132]. Subsequent studies have indeed shown low and very variable but detectable levels of gene flow in collinear regions at different loci in both directions [6,133–135]. A further and somewhat striking piece of evidence in favour of ongoing gene flow is found in mitochondrial DNA (mtDNA) which shares significant similarity between sympatric populations of *D. pseudoobscura* and *D. persimilis*. However, almost no mtDNA similarity is found between allopatric populations. This is puzzling because of the previously discussed high level of nuclear genomic divergence, but it suggests low levels of gene flow with selection pressures against nuclear gene introgression from backcrossing [136].

The interchromosomal effect, where low recombination in inverted regions within heterokaryotypes is compensated with a higher crossing over rate in non-inverted regions, is seen in *D. pseudoobscura* [137]. The interchromosomal effect within F_1_ heterokaryotype hybrids is also correlated with higher levels of noncrossover gene conversion *within* inverted regions in laboratory crosses. Species barriers established by inversions may therefore be dampened in F_1_ hybrids where gene flow can potentially occur from one parental karyotype to the other via backcrossing. As has been previously established, backcrossing from hybrids is extremely rare in nature, but if it were to occur then this has the potential to introgress genetic material between species [138] although in reality it is unlikely that gene flow across inverted regions is occurring commonly enough, if at all, in natural populations to create any tangible weakening of species barriers [113].

There are many models and approaches to tackle the questions of gene flow between species and our current estimates appear varied and wide ranging. However, with developing techniques it should hopefully be possible to gain better resolution going forward as to how much gene flow currently does occur between natural populations and whether collinear regions of the genome are still experiencing gene flow.

### Genetic mechanisms of post-zygotic reproductive isolation

Building on Lancefield’s work, Dobzhansky and collaborators produced a series of papers on reproductive isolation between these pairs, identifying a failure in spermatogenesis due to reduced testes size [139]. Corroborating the results of Lancefield, these phenotypes were associated with chromosomal incompatibilities between the X chromosome of one ‘race’ and the autosomes of the other and also identified to be polygenic, with the genes on these incompatible chromosomes being additive in nature [103,140]. This is an example of what is now known as the Bateson-Dobzhansky-Muller model; that hybrid dysgenesis is caused by negative epistatic interactions between different loci in the different species. This model can help explain low hybrid fitness at both pre-mating and post-mating levels. For example, F_1_ males are less vigorous in their courtship, with hybrids consisting of a *D. persimilis* X chromosome within a *D. pseudoobscura* autosomal background being particularly weak to court [56]. Orr found a strong genetic basis to post-zygotic isolation due to incompatibilities between loci on interspecific X and Y chromosomes [141], although this work placed less emphasis on the role of X-autosomal epistasis than previous studies. Orr also found a minor role for maternal effects in semi-sterility; X-cytoplasmic incompatibilities appeared to affect the fertility of female offspring resulting from female F_1_ backcrosses to parental species.

Over time it has been increasingly appreciated just how complex these epistatic interactions are. They have been shown to be highly polygenic, with multiple specific QTLs needed to initiate a response [142,143]. These epistatic interactions also go beyond simple additive or dominant-recessive QTL paradigms, with loci appearing to be dominant or recessive depending on which other loci they interact with. This work by Chang et al. specifically crossed *D. persimilis* with *D. p. bogotana* rather than the more widely used, and directly comparable, *D. p. pseudoobscura*. Therefore, their results technically infer an indirect explanation regarding general models of *D. pseudoobscura* and *D. persimilis* hybrid dysfunction but the mechanisms seem likely to be similar. Such complexity implies that there are many more loci involved in hybrid sterility than might have initially been thought but are difficult to detect. It also suggests that the molecular mechanisms of these genetic interactions in producing hybrid dysgenesis are equally complicated.

A prominent hypothesis for the underlying mechanism of the Bateson-Dobzhansky-Muller model states that misexpression of genes within an interspecific background could result in observed hybrid disfunction [144]. Support for this between *D. pseudoobscura* and *D. persimilis* was initially limited, as early studies did not find any misexpression in tested transcripts [145]. However, an antisense transcript orthologous to the *D. melanogaster* gene *TRAP100* was later found that did support the misexpression hypothesis. This transcript is underexpressed in F_1_ hybrids and is implicated in regulating *TRAP100* gene expression from the sense strand. *TRAP100* itself is a highly conserved gene with an important role in transcription [146], thus F_1_ hybrids may misexpress *TRAP100* in the absence of proper gene regulation, to detrimental effects of fertility [62]. Later, it was found that male specific genes are also underexpressed in hybrid males of *D. simulans* and *D. mauritiana* [147]. Three genes from this study were tested in *D. pseudoobscura* and *D. persimilis*: Acylphosphatase (*Acyp*), always early (*aly*) and CG5762, all of which are found on the X chromosome. *aly* is involved in the spermiogenesis pathway while CG5762 is expressed in the testes, therefore they were candidates for involvement in hybrid sterility. Expression analysis of these genes in F_1_ hybrids showed that the *D. persimilis* copies of all three and the *D. pseudoobscura* copies of *Acyp* and *aly* were significantly underexpressed in F_1_ hybrid males [63] (Figure 3). Such work supports the misexpression hypothesis. However, due to the complexity of the epistatic interactions, much more work will be needed to map out the landscape of epistasis and the various networks of misexpression between the implicated loci.

Finally, non-coding RNAs (ncRNAs) show significant differential expression between *D. pseudoobscura* and *D. persimilis*. ncRNAs have very limited protein coding function but are associated with regulation of processes such as cell functioning, dosage compensation, and fertility [148,149]. We have a nascent understanding of ncRNAs, especially in the context of species divergence but this does at least demonstrate how quickly expression differences can evolve between species and cause negative epistasis in the ‘wrong’ genomic background [150].

Currently, our knowledge of the genetics of reproductive isolation between *D. persimilis* and *D. pseudoobscura* prominently concerns analyses of QTLs influencing sterility residing in inversions. These QTLs, which typically act epistatically with other genes during normal development of fertility, are incompatible in an interspecific background. This may be due to misexpression which leads to poor hybrid fertility; however, the mechanistic basis of how such genes influence reproductive isolation needs much more work. Karyotypic differences between populations that speciated into *D. pseudoobscura* and *D. persimilis* and the evolution of incompatible genes on these inversions offer a test case for the emergence of reproductive isolation. Once reproductive isolation was established and significant fitness costs arose from hybrid mating in secondary contact, it seems possible and even likely that reinforcement would be favoured to avoid this as a response to post mating incompatibilities. However, despite over a hundred years of research, there are still many questions to answer about the causes of speciation between these species.

## *Reproductive isolation between* D. pseudoobscura bogotana *and* D. pseudoobscura pseudoobscura

One common issue in studies of speciation is that, when studying two reproductively isolated species, it is difficult to infer key changes which contributed to speciation versus differences which accumulate after speciation. Hence many studies concentrate on differentiated populations or subspecies, as these may be more likely to represent changes during the key early stages of speciation. *D. pseudoobscura* has also been studied in this context as it consists of two subspecies that show significant pre-zygotic and post-zygotic isolation [151]. They are allopatric and were thought to have started diverging as recently as ~ 150,000 years ago [138,152]. *D. p. bogotana* is endemic to Bogotá, Colombia, separate from any populations of *D. p. pseudoobscura*, and this smaller population range means that *D. p. bogotana* exhibit significantly less genetic polymorphism than *D. p. pseudoobscura* [153] yet contain structural variants on the 3^rd^ chromosome that are found only very rarely in *D. p. pseudoobscura* [154]. There is significant asymmetric reproductive isolation between the two subspecies at pre-mating, PMPZ, and post-zygotic levels. *D. p. bogotana* females will mate with a male of either subspecies, whereas *D. p. pseudoobscura* females prefer consubspecific males. Courtship is also different between the two, with each subspecies producing a unique CHC profile and *D. p. bogotana* males courting more slowly than *D. p. pseudoobscura* males towards females of either subspecies [155].

As both subspecies must, by definition, be allopatric, no sympatric populations are available to compare against for signs of pre-mating reinforcement. However, PMPZ isolation occurs in the form of CSP, similar to that seen between *D. pseudoobscura* and *D. persimilis*. The effect size is slight but significant; in tests of sperm competition, consubspecific males sire more offspring than heterosubspecific males [156].

*D*. *p. pseudoobscura* males crossed to *D. p. bogotana* females produce typically sterile males whereas the reciprocal cross results in fertile males [157,158]. Like between *D. pseudoobscura* and *D. persimilis* this unidirectional ‘sterility’ appears to be due to the Bateson-Dobzhansky-Muller model through negative chromosomal epistasis and maternal effects [159,160]. Also like between *D. pseudoobscura* and *D. persimilis*, hybrid sterility is found to be associated with gene misexpression [161]. Genes identified in the negative epistasis of the ‘sterile’ F_1_ males consist of cell adhesion genes and proteases, which are prominent components of the reproductive system and are involved in fertility [162]. One key gene, named *Overdrive* (*Ovd*) [163], crucially appears insufficient by itself in producing hybrid male sterility, requiring other specific loci to interact with [164–166]. A network of over 50 genes have been found that link to *Ovd* to producing trans-regulatory effects on the expression of genes within that network [64] (Figure 3). *Ovd* is highly diverged between *D. p. pseudoobscura* and *D. p. bogotana* as evidenced by a large number of fixed amino acid changes, and this divergence causes misexpression in the gene network within F_1_ hybrid males. Specifically, *Ovd* appears to be affecting protease expression which in turn is important for proper formation of sperm. Curiously, low levels of fertility can be rescued in ‘sterile’ F_1_ males after being aged for several weeks [167]. When backcrossed, these older F_1_ males exhibit significant segregation distortion, producing almost 100% daughters, which is not observed in backcrosses of the fully fertile F_1_ males from the reciprocal cross. This effect has been localized to epistatic effects between several loci on the *D. p. bogotana* derived X chromosome which induce segregation distortion and is typically supressed by genes on *D. p. bogotana* autosomes. However, in a different subspecific background this segregation distortion is expressed. These studies, therefore, provide strong evidence that *Ovd* is a major component of post-reproductive isolation between *D. p. pseudoobscura* and *D. p. bogotana*, as well as supporting the idea that epistatic interactions resulting in hybrid dysgenesis is a highly polygenic and complex landscape.

Typically, experiments between *D. pseudoobscura* and *D. persimilis* have used the *D. p. pseudoobscura* subspecies because of their shared range with *D. persimilis* which make it ideal for work on reinforcement. These subspecies allow for an extension of this model in multiple ways. First, contrasts between *D. persimilis* and the two subspecies of *D. pseudoobscura* offer comparisons of speciation between allopatric and parapatric populations. Secondly, reproductive isolation and genetic differences between *D. p. pseudoobscura* and *D. p. bogotana* make an important, interesting and younger model pair for testing mechanisms of reinforcement, PMPZ isolation and underlying post-zygotic genetic bases.

## Conclusions

*D*. *pseudoobscura* and *D. persimilis* continue to be a valuable model system for studying speciation and reproductive isolation. Key research areas over the last 100 years have focused on reproductive isolation through the lens of pre-mating and PMPZ mechanisms of isolation, reinforcement of these mechanisms to increase isolation, and the underlying genetic causes focusing on the role of inversions and identification of speciation genes.

There does appear to be solid evidence for pre-zygotic reinforcement between this species, although not without some controversy [168]. While reinforcement is an attractive model for promoting reproductive isolation, clear examples are limited and with this system evidence is hampered by suggestions of fitness costs to reinforcement, potentially low selective pressure and potential alternative explanations such as divergence by sexual selection. This pair has been a crucial system for furthering our understanding of post-zygotic isolation with research revealing how unique structural variants can counter interspecies gene flow due to suppressed recombination, promoting the conditions needed for hybrid incompatibility genes to evolve and fix within lineages that contribute further to speciation. Furthermore, studies investigating the genetic basis for hybrid dysgenesis have shown support for the Bateson-Dobzhansky-Muller model and provided a mechanical understanding of the negative epistasis that results in misexpression of key regulatory genes. We have also briefly described studies from two subspecies of *D. pseudoobscura* that show evidence of reproductive isolation through both pre- and post-zygotic mechanisms. These subspecies could themselves be said to be undergoing speciation and are likely to provide an additional model for how species divergence forms at an earlier evolutionary state than *D. pseudoobscura* and *D. persimilis*.

Despite a century of study, many questions in this system remain outstanding and exciting new technological advances allow us to tackle them. With regards to pre-zygotic isolation modern proteomic analysis can characterize sperm types and the ejaculate composition for *D. pseudoobscura* and *D. persimilis* to generate a mechanistic basis for CSP. In terms of pre-mating isolation, we would like to know more precisely which courtship cues females use to decide which male to accept for mating, how these differ between populations and whether these are under reinforcing selection. Reinforcement itself appears costly but we do not know what these costs are. Modern sequencing and analytical techniques can provide us with a much higher resolution regarding how much gene flow is occurring between natural populations currently, if at all. Finally, the budding reproductive isolation between *D. p. pseudoobscura* and *D. p bogotana* and comparisons to *D. pseudoobscura* and *D. persimilis* are likely to provide a wealth of data and may be a vital tool to exploring the early stages of speciation and reproductive isolation between them.

[169–172]

## Data Availability

No new data was generated for this study.

## References

[1] Sturtevant AH. Genetic studies on *Drosophila simulans*. I. Introduction. Hybrids with *Drosophila melanogaster*. Genetics. 1920;5(5):488–22. doi: 10.1093/genetics/5.5.488 17245951 PMC1200491

[2] Barbash DA. Ninety years of *Drosophila melanogaster* hybrids. Genetics. 2010;186(1):1–8. doi: 10.1534/genetics.110.121459 20855573 PMC2940278

[3] Lancefield DE. A genetic study of crosses of two races or physiological species of *Drosophila obscura Z*. Ver-erbungslehre. 1929;52(1):287–317. doi: 10.1007/BF01847272

[4] Cowell F. 100 years of Haldane’s rule. J Evol Biol. 2023;36(2):337–346. doi: 10.1111/jeb.14112 36357993 PMC10098713

[5] Haldane JBS. Sex ratio and unisexual sterility in hybrid animals. Journ Gen. 1922;12(2):101–109. doi: 10.1007/BF02983075

[6] Korunes KL, Machado CA, Noor MAF. Inversions shape the divergence of *Drosophila pseudoobscura* and *Drosophila persimilis* on multiple timescales. Evolution. 2021;75(7):1820–1834. doi: 10.1111/evo.14278 34041743

[7] Myers EM, Harwell TI, Yale EL, et al. Multifaceted, cross-generational costs of hybridization in sibling *Drosophila* species. PLOS ONE. 2013;8(11):e80331. doi: 10.1371/journal.pone.0080331 24265807 PMC3827178

[8] Kayserili MA, Gerrard DT, Tomancak P, et al. An excess of gene expression divergence on the x chromosome in *Drosophila* embryos: implications for the faster-x hypothesis. PLOS Genet. 2012;8(12):e1003200. doi: 10.1371/journal.pgen.1003200 23300473 PMC3531489

[9] Rizki MTM. Morphological differences between two sibling species, *Drosophila pseudoobscura* and *Drosophila persimilis*. Proc Natl Acad Sci. 1951;37(3):156–159. doi: 10.1073/pnas.37.3.156 14808171 PMC1063323

[10] Dobzhansky T. Genetics of natural populations. XVI. Altitudinal and seasonal changes produced by natural selection in certain populations of *Drosophila pseudoobscura* and *Drosophila persimilis*. Genetics. 1948;33(2):158–176. doi: 10.1093/genetics/33.2.158 18856563 PMC1209402

[11] Dobzhansky T. Genetics of natural populations. XX. changes induced by drought in *Drosophila pseudoobscura* and *Drosophila persimilis*. Evolution. 1952;6(2):234–243. doi: 10.2307/2405627

[12] Prakash S. Genetic divergence in closely related sibling species *Drosophila pseudoobscura*, *Drosophila persimilis* and *Drosophila miranda*. Evolution. 1977;31(1):14–23. doi: 10.1111/j.1558-5646.1977.tb00977.x 28567734

[13] Dobzhansky T, Epling C. Contributions to the genetics, taxonomy, and ecology of *Drosophila pseudoobscura* and its relatives. Annals of the Entomological Society of America. 1946;39(1): doi: 10.1093/aesa/39.1.151

[14] Klaczko LB, Taylor CE, Powell JR. Genetic variation for dispersal by *Drosophila pseudoobscura* and *Drosophila persimilis*. Genetics. 1986;112(2):229–235. doi: 10.1093/genetics/112.2.229 17246313 PMC1202698

[15] Spassky B, Dobzhansky T. Responses of various strains of *Drosophila pseudoobscura* and *Drosophila persimilis* to light and to gravity. Am Nat. 1967;101(917):59–63. doi: 10.1086/282469

[16] Wogaman DJ, Seiger MB. Light intensity as a factor in the choice of an oviposition site by *Drosophila pseudoobscura* and *Drosophila persimilis*. Can J Genet Cytol. 1983;25(4):370–377. doi: 10.1139/g83-057

[17] Westram AM, Stankowski S, Surendranadh P, et al. What is reproductive isolation? J Evol Biol. 2022;35(9):1143–1164. doi: 10.1111/jeb.14005 36063156 PMC9542822

[18] Jin B, Barbash DA, Castillo DM. Divergent selection on behavioural and chemical traits between reproductively isolated populations of *Drosophila melanogaster*. J Evol Biol. 2022;35(5):693–707. doi: 10.1111/jeb.14007 35411988 PMC9320809

[19] Rundle HD, Chenoweth SF, Doughty P, et al. Divergent selection and the evolution of signal traits and mating preferences. PLOS Biol. 2005;3(11):e368. doi: 10.1371/journal.pbio.0030368 16231971 PMC1262626

[20] Safran RJ, Scordato ESC, Symes LB, et al. Contributions of natural and sexual selection to the evolution of premating reproductive isolation: a research agenda. Trends Ecol Evol. 2013;28:643–650. doi: 10.1016/j.tree.2013.08.004 24054911

[21] Blair WF. Mating call and stage of speciation in the *Microhyla olivacea*—*M. carolinensis* complex. Evolution. 1955;9(4):469–480. doi: 10.1111/j.1558-5646.1955.tb01556.x

[22] Butlin RK. Speciation by reinforcement. Trends Ecol Evol. 1987;2(1):8–13. doi: 10.1016/0169-5347(87)90193-5 21227808

[23] Servedio MR, Noor MAF. The role of reinforcement in speciation: theory and data. Annu Rev Ecol Evol Syst. 2003;34:339–364. doi: 10.1146/annurev.ecolsys.34.011802.132412

[24] Garlovsky MD, Whittington E, Albrecht T, et al. Synthesis and scope of the role of postmating prezygotic isolation in speciation. Cold Spring Harb Perspect Biol. 2024;16(10):a041429. doi: 10.1101/cshperspect.a041429 38151330 PMC11444258

[25] Orr HA, Presgraves DC. Speciation by postzygotic isolation: forces, genes and molecules. Bioessays. 2000;22(12):1085–1094. doi: 10.1002/1521-1878(200012)22:12<1085::AID-BIES6>3.0.CO;2-G 11084624

[26] Kulmuni J, Butlin RK, Lucek K, et al. Towards the completion of speciation: the evolution of reproductive isolation beyond the first barriers. Phil Trans R Soc B: Biol Sci. 2020;375(1806):20190528. doi: 10.1098/rstb.2019.0528 PMC7423269 32654637

[27] Levene H, Dobzhansky T. Experiments on sexual isolation in *Drosophila*. V. the effect of varying proportions of *Drosophila pseudoobscura* and *Drosophilia persimilis* on the frequency of insemination in mixed populations. Proc Natl Acad Sci. 1945;31(9):274–281. doi: 10.1073/pnas.31.9.274 16578166 PMC1078821

[28] Mayr E, Dobzhansky T. Experiments on sexual isolation in *Drosophila*. IV. Modification of the degree of isolation between *Drosophila pseudoobscura* and *Drosophila persimilis* and of sexual preferences in *Drosophila prosaltans*. Proc Natl Acad Sci USA. 1945;31(2):75–82. doi: 10.1073/pnas.31.2.75 16588688 PMC1078756

[29] Dobzhansky T. Experiments on sexual isolation in *Drosophila*. X. Reproductive isolation between *Drosophila pseudoobscura* and *Drosophila persimilis* under natural and under laboratory conditions. Proc Natl Acad Sci USA. 1951;37(12):792–796. doi: 10.1073/pnas.37.12.792 16589029 PMC1063472

[30] Mayr E. Experiments on sexual isolation in *Drosophila*. VI. Isolation between *Drosophila pseudoobscura* and *Drosophila persimilis* and their hybrids. Proc Natl Acad Sci U S A. 1946;32(3):57–59. doi: 10.1073/pnas.32.3.57 16578193 PMC1078879

[31] Mayr E. Experiments on sexual isolation in *Drosophila*. VII. The nature of the isolating mechanisms between *Drosophila pseudoobscura* and *Drosophila persimilis*. Proc Natl Acad Sci USA. 1946;32(5):128–137. doi: 10.1073/pnas.32.5.128 16588725 PMC1078899

[32] Krasovec M. The spontaneous mutation rate of *Drosophila pseudoobscura*. G3. 2021;11(7):jkab151. doi: 10.1093/g3journal/jkab151 33950174 PMC8495931

[33] Sharp NP, Agrawal AF. Evidence for elevated mutation rates in low-quality genotypes. Proc Natl Acad Sci. 2012;109(16):6142–6146. doi: 10.1073/pnas.1118918109 22451943 PMC3341077

[34] Yang S, Wang L, Huang J, et al. Parent–progeny sequencing indicates higher mutation rates in heterozygotes. Nature. 2015;523(7561):463–467. doi: 10.1038/nature14649 26176923

[35] Wiley C, Qvarnström A, Andersson G, et al. Postzygotic isolation over multiple generations of hybrid descendents in a natural hybrid zone: how well do single-generation estimates reflect reproductive isolation? Evolution. 2009;63(7):1731–1739. doi: 10.1111/j.1558-5646.2009.00674.x 19245675

[36] Koopman KF. Natural selection for reproductive isolation between *Drosophila pseudoobscura* and *Drosophila persimilis*. Evolution. 1950;4(2):135–148. doi: 10.2307/2405390

[37] Kessler S. Selection for and against ethological isolation between *Drosophila pseudoobscura* and *Drosophila persimilis*. Evolution. 1966;20(4):634. doi: 10.2307/2406597 28562900

[38] Rice WR, Hostert EE. Laboratory experiments on speciation: what have we learned in 40 years? Evolution. 1993;47(6):1637–1653. doi: 10.2307/2410209 28568007

[39] Kirkpatrick M, Servedio MR. The reinforcement of mating preferences on an island. Genetics. 1999;151(2):865–884. doi: 10.1093/genetics/151.2.865 9927476 PMC1460501

[40] Merrell DJ. Sexual isolation between *Drosophila persimilis* and *Drosophila pseudoobscura*. Am Nat. 1954;88(839):93–99. doi: 10.1086/281814

[41] Noor MAF. Absence of species discrimination in and males. Anim Behaviour. 1996;52(6):1205–1210. doi: 10.1006/anbe.1996.0268

[42] Noor MA. Speciation driven by natural selection in *Drosophila*. Nature. 1995;375(6533):674–675. doi: 10.1038/375674a0 7791899

[43] Butlin RK. Reinforcement: an idea evolving. Trends Ecol Evol. 1995;10(11):432–434. doi: 10.1016/S0169-5347(00)89173-9 21237095

[44] Schaeffer SW, Miller EL. Estimates of gene flow in *Drosophila pseudoobscura* determined from nucleotide sequence analysis of the alcohol dehydrogenase region. Genetics. 1992;132(2):471–480. doi: 10.1093/genetics/132.2.471 1427038 PMC1205150

[45] Schaeffer SW, Goetting-Minesky MP, Kovacevic M, et al. Evolutionary genomics of inversions in *Drosophila pseudoobscura*: evidence for epistasis. Proc Natl Acad Sci. 2003;100(14):8319–8324. doi: 10.1073/pnas.1432900100 12824467 PMC166227

[46] Lorch PD, Servedio MR. Postmating-prezygotic isolation is not an important source of selection for reinforcement within and between species in *Drosophila pseudoobscura* and *D. persimilis*. Evolution. 2005;59(5):1039–1045. doi: 10.1111/j.0014-3820.2005.tb01042.x 16136803

[47] Anderson WW, Kim Y-K. Sexual isolation between sympatric and allopatric populations of *Drosophila pseudoobscura* and *D. persimilis*. Behav Genet. 2005;35(3):305–312. doi: 10.1007/s10519-005-3222-3 15864445

[48] Noor MAF, Ortíz-Barrientos D. Simulating natural conditions in the laboratory: a re-examination of sexual isolation between sympatric and allopatric populations of *Drosophila pseudoobscura* and *D. persimilis*. Behav Genet. 2006;36(2):322–327. doi: 10.1007/s10519-005-9033-8 16502138

[49] Anderson WW, Kim Y-K. A further analysis of sexual isolation between sympatric and allopatric populations of *Drosophila pseudoobscura* and *D. persimilis*. Behav Genet. 2006;36(2):328–330. doi: 10.1007/s10519-005-9030-y 16477520

[50] Garner AG, Goulet BE, Farnitano MC, et al. Genomic signatures of reinforcement. Genes (Basel). 2018;9(4):191. doi: 10.3390/genes9040191 29614048 PMC5924533

[51] Bailey N, Ruiz C, Tosi A, et al. Genomic analysis of the rhesus macaque (*Macaca mulatta*) and the cynomolgus macaque (*Macaca fascicularis*) uncover polygenic signatures of reinforcement speciation. Ecol Evol. 2023;13(10):e10571. doi: 10.1002/ece3.10571 37849934 PMC10577069

[52] North HL, Caminade P, Severac D, et al. The role of copy-number variation in the reinforcement of sexual isolation between the two European subspecies of the house mouse. Phil Trans R Soc B: Biol Sci. 2020;375(1806):20190540. doi: 10.1098/rstb.2019.0540 PMC7423270 32654648

[53] Ospina OE, Lemmon AR, Dye M, et al. Neurogenomic divergence during speciation by reinforcement of mating behaviors in chorus frogs (*Pseudacris*). BMC Genomics. 2021;22(1):711. doi: 10.1186/s12864-021-07995-3 34600496 PMC8487493

[54] Laetsch DR, Bisschop G, Martin SH, et al. Demographically explicit scans for barriers to gene flow using gImble. PLOS Genet. 2023;19(10):e1010999. doi: 10.1371/journal.pgen.1010999 37816069 PMC10610087

[55] Brown RGB. Courtship behaviour in the *Drosophila obscura* group. I: *D. pseudoobscura*. Behaviour. 1964;23(1–2):61–105. doi: 10.1163/156853964X00094.

[56] Noor MAF. Genetics of sexual isolation and courtship dysfunction in male hybrids of *Drosophila pseudoobscura* and *Drosophila persimilis*. Evolution. 1997;51(3):809–815. doi: 10.2307/2411156 28568570

[57] Tan CC. Genetics of sexual isolation between *Drosophila pseudoobscura* and *Drosophila persimilis*. Genetics. 1946;31(6):558–573. doi: 10.1093/genetics/31.6.558 17247220 PMC1209354

[58] Ewing AW. The genetic basis of sound production in *Drosophila pseudoobscura* and *D. persimilis*. Anim Behaviour. 1969;17:555–560. doi: 10.1016/0003-3472(69)90164-X 5370966

[59] Williams MA, Blouin AG, Noor MAF. Courtship songs of *Drosophila pseudoobscura* and *D. persimilis*. II. Genetics of species differences. Heredity (Edinb). 2001;86(1):68–77. doi: 10.1046/j.1365-2540.2001.00811.x 11298817

[60] Hernández MV, Fabre CCG. Triggers of the postural display of courtship in *Drosophila persimilis* flies. J Insect Behav. 2017;30(5):582–594. doi: 10.1007/s10905-017-9641-1 29104367 PMC5656710

[61] Ortíz-Barrientos D, Counterman BA, Noor MAF, et al. The genetics of speciation by reinforcement. PLOS Biol. 2004;2(12):e416. doi: 10.1371/journal.pbio.0020416 15550988 PMC529318

[62] Noor MAF, Michalak P, Donze D. Characterization of a male-predominant antisense transcript underexpressed in hybrids of *Drosophila pseudoobscura* and *D. persimilis*. Genetics. 2003;165(4):1823–1830. doi: 10.1093/genetics/165.4.1823 14704168 PMC1462881

[63] Noor MAF. Patterns of evolution of genes disrupted in expression in *Drosophila* species hybrids. Genet Res. 2005;85(2):119–125. doi: 10.1017/S0016672305007500 16174330

[64] Go AC, Civetta A. Divergence of X-linked *trans* regulatory proteins and the misexpression of gene targets in sterile *Drosophila pseudoobscura* hybrids. BMC Genomics. 2022;23(1):30. doi: 10.1186/s12864-021-08267-w 34991488 PMC8740060

[65] Barnwell CV, Noor MAF. Failure to replicate two mate preference QTLs across multiple strains of *Drosophila pseudoobscura*. J Hered. 2008;99(6):653–656. doi: 10.1093/jhered/esn069 18728083 PMC2574947

[66] Laturney M, Moehring AJ. The genetic basis of female mate preference and species isolation in *Drosophila*. Int J Evol Biol. 2012;2012:1–13. doi: 10.1155/2012/328392 PMC3432541 22957299

[67] Kandul NP, Wright KM, Kandul EV, et al. No evidence for learned mating discrimination in male *Drosophila pseudoobscura*. BMC Evol Biol. 2006;6(1):54. doi: 10.1186/1471-2148-6-54 16824212 PMC1534054

[68] Dukas R. Learning decreases heterospecific courtship and mating in fruit flies. Biol Lett. 2008;4(6):645–647. doi: 10.1098/rsbl.2008.0437 18842572 PMC2614174

[69] Dukas R. Dynamics of learning in the context of courtship in *Drosophila persimilis* and *D. pseudoobscura*. Anim Behaviour. 2009;77(1):253–259. doi: 10.1016/j.anbehav.2008.10.010

[70] Olofsson H, Frame AM, Servedio MR. Can reinforcement occur with a learned trait? Evolution. 2011;65(7):1992–2003. doi: 10.1111/j.1558-5646.2011.01286.x 21729054

[71] Dobzhansky T. Effectiveness of intraspecific and interspecific matings in *Drosophila pseudoobscura* and *Drosophila persimilis*. Am Nat. 1947;81:66–72. doi: 10.1086/281502 20286615

[72] Thornhill R. Cryptic female choice and its implications in the scorpionfly *Harpobittacus nigriceps*. Am Nat. 1983;122(6):765–788. doi: 10.1086/284170

[73] Firman RC, Gasparini C, Manier MK, et al. Postmating female control: 20 years of cryptic female choice. Trends Ecol Evol. 2017;32(5):368–382. doi: 10.1016/j.tree.2017.02.010 28318651 PMC5511330

[74] Comeault AA, Venkat A, Matute DR. Correlated evolution of male and female reproductive traits drive a cascading effect of reinforcement in *Drosophila yakuba*. Proc R Soc B: Biol Sci. 2016;283(1835):20160730. doi: 10.1098/rspb.2016.0730 PMC4971202 27440664

[75] Matute DR, Barton NH. Reinforcement of gametic isolation in *Drosophila*. PLOS Biol. 2010;8(3):e1000341. doi: 10.1371/journal.pbio.1000341 20351771 PMC2843595

[76] Poikela N, Kinnunen J, Wurdack M, et al. Strength of sexual and postmating prezygotic barriers varies between sympatric populations with different histories and species abundances. Evolution. 2019;73(6):1182–1199. doi: 10.1111/evo.13732 30957216

[77] Castillo DM, Moyle LC. Conspecific sperm precedence is reinforced, but postcopulatory sexual selection weakened, in sympatric populations of *Drosophila*. Proc R Soc B: Biol Sci. 2019;286(1899):20182535. doi: 10.1098/rspb.2018.2535 PMC6452082 30900533

[78] Holman L, Snook RR. Spermicide, cryptic female choice and the evolution of sperm form and function. J Evol Biol. 2006;19(5):1660–1670. doi: 10.1111/j.1420-9101.2006.01112.x 16910995

[79] Cook PA, Wedell N. Non-fertile sperm delay female remating. Nature. 1999;397(6719):486–486. doi: 10.1038/17257

[80] Alpern JHM, Asselin MM, Moehring AJ. Identification of a novel sperm class and its role in fertilization in *Drosophila*. J Evol Biol. 2019;32(3):259–266. doi: 10.1111/jeb.13409 30484924

[81] Holman L, Snook RR. A sterile sperm caste protects brother fertile sperm from female-mediated death in *Drosophila pseudoobscura*. Curr Biol. 2008;18(4):292–296. doi: 10.1016/j.cub.2008.01.048 18291649

[82] Peckenpaugh B, Castillo DM, Moyle LC. Testing potential mechanisms of conspecific sperm precedence in *Drosophila pseudoobscura*. J Evol Biol. 2021;34(12):1970–1980. doi: 10.1111/jeb.13946 34653290 PMC10889848

[83] Heifetz Y, Tram U, Wolfner MF. Male contributions to egg production: the role of accessory gland products and sperm in *Drosophila melanogaster*. Proc R Soc Lond B. 2001;268(1463):175–180. doi: 10.1098/rspb.2000.1347 PMC1088588 11209888

[84] Kalb JM, DiBenedetto AJ, Wolfner MF. Probing the function of *Drosophila melanogaster* accessory glands by directed cell ablation. Proc Natl Acad Sci. 1993;90(17):8093–8097. doi: 10.1073/pnas.90.17.8093 8367469 PMC47294

[85] Lung O, Tram U, Finnerty CM, et al. The *Drosophila melanogaster* seminal fluid protein Acp62F is a protease inhibitor that is toxic upon ectopic expression. Genetics. 2002;160(1):211–224. doi: 10.1093/genetics/160.1.211 11805057 PMC1461949

[86] Hopkins BR, Perry JC. The evolution of sex peptide: sexual conflict, cooperation, and coevolution. Biol Rev Camb Philos Soc. 2022;97(4):1426–1448. doi: 10.1111/brv.12849 35249265 PMC9256762

[87] Davis JS, Castillo DM, Moyle LC. Remating responses are consistent with male postcopulatory manipulation but not reinforcement in *D. pseudoobscura*. Ecol Evol. 2017;7(2):507–515. doi: 10.1002/ece3.2628 28116047 PMC5243186

[88] Yukilevich R, Aoki F. Is cascade reinforcement likely when sympatric and allopatric populations exchange migrants? Curr Zool. 2016;62(2):155–167. doi: 10.1093/cz/zow007 29491903 PMC5804230

[89] Myers EM, Frankino WA, Etges WJ. Time in a bottle: the evolutionary fate of species discrimination in sibling *Drosophila* species. PLOS ONE. 2012;7(2):e31759. doi: 10.1371/journal.pone.0031759 22384069 PMC3288057

[90] Haerty W, Jagadeeshan S, Kulathinal RJ, et al. Evolution in the fast lane: rapidly evolving sex-related genes in *Drosophila*. Genetics. 2007;177(3):1321–1335. doi: 10.1534/genetics.107.078865 18039869 PMC2147986

[91] Promislow DEL, Jung CF, Arnold ML. Age-specific fitness components in hybrid females of *Drosophila pseudoobscura* and *D. persimilis*. J Heredity. 2001;92(1):30–37. doi: 10.1093/jhered/92.1.30 11336226

[92] Lande R. Models of speciation by sexual selection on polygenic traits. Proc Natl Acad Sci. 1981;78(6):3721–3725. doi: 10.1073/pnas.78.6.3721 16593036 PMC319643

[93] Shaw KL, Cooney CR, Mendelson TC, et al. How important is sexual isolation to speciation? Cold Spring Harb Perspect Biol. 2024;16:a041427. doi: 10.1101/cshperspect.a041427 38346859 PMC10982695

[94] West-Eberhard MJ. Sexual selection, social competition, and speciation. Q Rev Biol. 1983;58(2):155–183. doi: 10.1086/413215

[95] Ghosh S, Joshi A. Evolution of reproductive isolation as a by-product of divergent life-history evolution in laboratory populations of *Drosophila melanogaster*. Ecol Evol. 2012;2(12):3214–3226. doi: 10.1002/ece3.413 23301185 PMC3539013

[96] Korol A, Rashkovetsky E, Iliadi K, et al. *Drosophila* flies in “evolution canyon” as a model for incipient sympatric speciation. Proc Natl Acad Sci USA. 2006;103(48):18184–18189. doi: 10.1073/pnas.0608777103 17108081 PMC1838727

[97] Syed ZA, Chatterjee M, Samant MA, et al. Reproductive isolation through experimental manipulation of sexually antagonistic coevolution in *Drosophila melanogaster*. Sci Rep. 2017;7(1):3330. doi: 10.1038/s41598-017-03182-1 28611437 PMC5469766

[98] Debelle A, Ritchie MG, Snook RR. Sexual selection and assortative mating: an experimental test. J Evol Biol. 2016;29(7):1307–1316. doi: 10.1111/jeb.12855 26970522

[99] Debelle A, Ritchie MG, Snook RR. Evolution of divergent female mating preference in response to experimental sexual selection. Evolution. 2014;68(9):2524–2533. doi: 10.1111/evo.12473 24931497 PMC4262321

[100] Snook RR, Robertson A, Crudgington HS, et al. Experimental manipulation of sexual selection and the evolution of courtship song in *Drosophila pseudoobscura*. Behav Genet. 2005;35(3):245–255. doi: 10.1007/s10519-005-3217-0 15864440

[101] Immonen E, Snook RR, Ritchie MG. Mating system variation drives rapid evolution of the female transcriptome in *Drosophila pseudoobscura*. Ecol Evol. 2014;4(11):2186–2201. doi: 10.1002/ece3.1098 25360260 PMC4201433

[102] Reis M, Vieira CP, Lata R, et al. Origin and consequences of chromosomal inversions in the virilis group of *Drosophila*. Genome Biol Evol. 2018;10(12):3152–3166. doi: 10.1093/gbe/evy239 30376068 PMC6278893

[103] Dobzhansky T. Studies on hybrid sterility. II. Localization of sterility factors in *Drosophila pseudoobscura* hybrids. Genetics. 1936;21(2):113–135. doi: 10.1093/genetics/21.2.113 17246786 PMC1208664

[104] Berdan EL, Barton NH, Butlin R, et al. How chromosomal inversions reorient the evolutionary process. J Evol Biol. 2023;36(12):1761–1782. doi: 10.1111/jeb.14242 37942504

[105] Kirkpatrick M. How and why chromosome inversions evolve. PLOS Biol. 2010;8(9):e1000501. doi: 10.1371/journal.pbio.1000501 20927412 PMC2946949

[106] Fuller ZL, Koury SA, Phadnis N, et al. How chromosomal rearrangements shape adaptation and speciation: case studies in *Drosophila pseudoobscura* and its sibling species *Drosophila persimilis*. Mol Ecol. 2019;28(6):1283–1301. doi: 10.1111/mec.14923 30402909 PMC6475473

[107] Tan CC. Salivary gland chromosomes in the two races of *Drosophila pseudoobscura*. Genetics. 1935;20(4):392–402. doi: 10.1093/genetics/20.4.392 17246768 PMC1208620

[108] Coyne JA, Moore BC, Moore JA, et al. Temporal stability of third-chromosome inversion frequencies in *Drosophila persimilis* and *D. pseudoobscura*. Evolution. 1992;46(5):1558–1563. doi: 10.2307/2409959 28568997

[109] Wallace AG, Detweiler D, Schaeffer SW. Evolutionary history of the third chromosome gene arrangements of *Drosophila pseudoobscura* inferred from inversion breakpoints. Mol Biol Evol. 2011;28(8):2219–2229. doi: 10.1093/molbev/msr039 21339510

[110] Norman RA, Prakash S. Variation in activities of amylase allozymes associated with chromosome inversions in *Drosophila pseudoobscura, D. persimilis and D. miranda*. Genet. 1980;95(1):187–209. doi: 10.1093/genetics/95.1.187 PMC1214216 6159250

[111] Navarro A, Barton NH. Accumulating postzygotic isolation genes in parapatry: a new twist on chromosomal speciation. Evolution. 2003;57(3):447–459. doi: 10.1111/j.0014-3820.2003.tb01537.x 12703935

[112] Noor MAF, Grams KL, Bertucci LA, et al. Chromosomal inversions and the reproductive isolation of species. Proc Natl Acad Sci. 2001;98(21):12084–12088. doi: 10.1073/pnas.221274498 11593019 PMC59771

[113] Stevison LS, Hoehn KB, Noor MAF. Effects of inversions on within- and between-species recombination and divergence. Genome Biol Evol. 2011;3:830–841. doi: 10.1093/gbe/evr081 21828374 PMC3171675

[114] Carpinteyro-Ponce J, Machado CA, Vieira C. The complex landscape of structural divergence between the *Drosophila pseudoobscura* and *D. persimilis* genomes. Genome Biol Evol. 2024;16(3):evae047. doi: 10.1093/gbe/evae047 38482945 PMC10980976

[115] Brown KM, Burk LM, Henagan LM, et al. A test of the chromosomal rearrangement model of speciation in *Drosophila pseudoobscura*. Evolution. 2004;58(8):1856–1860. doi: 10.1111/j.0014-3820.2004.tb00469.x 15446438

[116] Kulathinal RJ, Stevison LS, Noor MAF, et al. The genomics of speciation in *Drosophila*: diversity, divergence, and introgression estimated using low-coverage genome sequencing. PLOS Genet. 2009;5(7):e1000550. doi: 10.1371/journal.pgen.1000550 19578407 PMC2696600

[117] Machado CA, Haselkorn TS, Noor MAF. Evaluation of the genomic extent of effects of fixed inversion differences on intraspecific variation and interspecific gene flow in *Drosophila pseudoobscura* and *D. persimilis*. Genetics. 2007;175(3):1289–1306. doi: 10.1534/genetics.106.064758 17179068 PMC1840060

[118] Noor MAF, Garfield DA, Schaeffer SW, et al. Divergence between the *Drosophila pseudoobscura* and *D. persimilis* genome sequences in relation to chromosomal inversions. Genetics. 2007;177(3):1417–1428. doi: 10.1534/genetics.107.070672 18039875 PMC2147956

[119] Ávila V, Marion de Procé S, Campos JL, et al. Faster-x effects in two *Drosophila* lineages. Genome Biol Evol. 2014;6(10):2968–2982. doi: 10.1093/gbe/evu229 25323954 PMC4224355

[120] McGaugh SE, Noor MAF. Genomic impacts of chromosomal inversions in parapatric *Drosophila* species. Philos Trans R Soc Lond B Biol Sci. 2012;367(1587):422–429. doi: 10.1098/rstb.2011.0250 22201171 PMC3233717

[121] Feder JL, Gejji R, Powell THQ, et al. Adaptive chromosomal divergence driven by mixed geographic mode of evolution. Evolution. 2011;65(8):2157–2170. doi: 10.1111/j.1558-5646.2011.01321.x 21790566

[122] Fuller ZL, Leonard CJ, Young RE, et al. Ancestral polymorphisms explain the role of chromosomal inversions in speciation. PloS Genet. 2018;14(7):e1007526. doi: 10.1371/journal.pgen.1007526 30059505 PMC6085072

[123] Lohse K, Clarke M, Ritchie MG, et al. Genome-wide tests for introgression between cactophilic *Drosophila* implicate a role of inversions during speciation. Evolution. 2015;69(5):1178–1190. doi: 10.1111/evo.12650 25824653 PMC5029762

[124] van Valen L. Introgression in laboratory populations of *Drosophila persimilis* and *D. pseudoobscura*. Heredity (Edinb). 1963;18(2):205–214. doi: 10.1038/hdy.1963.22

[125] Wu CI, Beckenbach AT. Evidence for extensive genetic differentiation between the sex-ratio and the standard arrangement of *Drosophila pseudoobscura* and *D. persimilis* and identification of hybrid sterility factors. Genetics. 1983;105(1):71–86. doi: 10.1093/genetics/105.1.71 17246158 PMC1202152

[126] Dobzhansky T. Is there gene exchange between *Drosophila pseudoobsura* and *Drosophila persimilis* in their natural habitats? Am Nat. 1973;107(954):312–314. doi: 10.1086/282833

[127] Kulathinal RJ, Singh RS. A biogeographic genetic approach for testing the role of reinforcement: the case of *Drosophila pseudoobscura* and *D. persimilis*. Evolution. 2000;54(1):210–217. doi: 10.1111/j.0014-3820.2000.tb00021.x 10937197

[128] Kulathinal RJ, Singh RS. Reinforcement with gene flow? A reply. Evolution. 2000;54(6):2176. doi: 10.1554/0014-3820(2000)054[2176:RWGFAR]2.0.CO;2

[129] Noor MAF, Johnson NA, Hey J. Gene flow between *Drosophila pseudoobscura* and *D. persimilis*. Evolution. 2000;54(6):2174–2175. doi: 10.1111/j.0014-3820.2000.tb01262.x 11209795

[130] Wang RL, Hey J. The speciation history of *Drosophila pseudoobscura* and close relatives: inferences from DNA sequence variation at the period locus. Genetics. 1996;144(3):1113–1126. doi: 10.1093/genetics/144.3.1113 8913754 PMC1207605

[131] Wang RL, Wakeley J, Hey J. Gene flow and natural selection in the origin of *Drosophila pseudoobscura* and close relatives. Genetics. 1997;147(3):1091–1106. doi: 10.1093/genetics/147.3.1091 9383055 PMC1208236

[132] Noor MAF, Grams KL, Bertucci LA, et al. The genetics of reproductive isolation and the potential for gene exchange between *Drosophila pseudoobscura* and *D. persimilis* via backcross hybrid males. Evolution. 2001;55(3):512–521. doi: 10.1554/0014-3820(2001)055[0512:TGORIA]2.0.CO;2 11327159

[133] Hey J, Nielsen R. Multilocus methods for estimating population sizes, migration rates and divergence time, with applications to the divergence of *Drosophila pseudoobscura* and *D. persimilis*. Genetics. 2004;167(2):747–760. doi: 10.1534/genetics.103.024182 15238526 PMC1470901

[134] Machado CA, Kliman RM, Markert JA, et al. Inferring the history of speciation from multilocus DNA sequence data: the case of *Drosophila pseudoobscura* and close relatives. Mol Biol Evol. 2002;19(4):472–488. doi: 10.1093/oxfordjournals.molbev.a004103 11919289

[135] Machado CA, Hey J. The causes of phylogenetic conflict in a classic *Drosophila* species group. Proc R Soc Lond Ser B Biol Sci. 2003;270(1520):1193–1202. doi: 10.1098/rspb.2003.2333 PMC1691361 12816659

[136] Powell JR. Interspecific cytoplasmic gene flow in the absence of nuclear gene flow: evidence from *Drosophila*. Proc Natl Acad Sci USA. 1983;80(2):492–495. doi: 10.1073/pnas.80.2.492 6300849 PMC393404

[137] Noor MAF, Smith KR. Recombination, statistical power, and genetic studies of sexual isolation in *Drosophila*. J Heredity. 2000;91(2):99–103. doi: 10.1093/jhered/91.2.99 10768121

[138] Korunes KL, Noor MAF. Pervasive gene conversion in chromosomal inversion heterozygotes. Mol Ecol. 2019;28(6):1302–1315. doi: 10.1111/mec.14921 30387889 PMC6475484

[139] Dobzhansky T. On the sterility of the interracial hybrids in *Drosophila pseudoobscura*. Proc Natl Acad Sci USA. 1933;19(4):397–403. doi: 10.1073/pnas.19.4.397 16577530 PMC1086012

[140] Dobzhansky T. Role of the autosomes in the *Drosophila pseudoobscura* hybrids. Proc Natl Acad Sci USA. 1933;19:950–953. doi: 10.1073/pnas.19.11.950 16577590 PMC1086257

[141] Orr HA. Genetics of male and female sterility in hybrids of *Drosophila pseudoobscura* and *D. persimilis*. Genetics. 1987;116(4):555–563. doi: 10.1093/genetics/116.4.555 3623079 PMC1203168

[142] Chang AS, Bennett SM, Noor MAF, et al. Epistasis among *Drosophila persimilis* factors conferring hybrid male sterility with *D. pseudoobscura bogotana*. PLOS ONE. 2010;5(10):e15377. doi: 10.1371/journal.pone.0015377 21060872 PMC2965152

[143] Chang AS, Noor MAF. Epistasis modifies the dominance of loci causing hybrid male sterility in the *Drosophila pseudoobscura* species group. Evolution. 2010;64(1):253–260. doi: 10.1111/j.1558-5646.2009.00823.x 19686263 PMC2827646

[144] Mack KL, Nachman MW. Gene regulation and speciation. Trends Genet. 2017;33(1):68–80. doi: 10.1016/j.tig.2016.11.003 27914620 PMC5182078

[145] Reiland J, Noor MA. Little qualitative RNA misexpression in sterile male F1 hybrids of *Drosophila pseudoobscura* and *D. persimilis*. BMC Evol Biol. 2002;2(1):16. doi: 10.1186/1471-2148-2-16 12223116 PMC126258

[146] Ito M, Okano HJ, Darnell RB, et al. The TRAP100 component of the TRAP/Mediator complex is essential in broad transcriptional events and development. Embo J. 2002;21(13):3464–3475. doi: 10.1093/emboj/cdf348 12093747 PMC126097

[147] Michalak P, Noor MAF. Genome-wide patterns of expression in *Drosophila* pure species and hybrid males. Mol Biol Evol. 2003;20(7):1070–1076. doi: 10.1093/molbev/msg119 12777520

[148] Jiang Z-F, Croshaw DA, Wang Y, et al. Enrichment of mRNA-like noncoding RNAs in the divergence of *Drosophila* males. Mol Biol Evol. 2011;28(4):1339–1348. doi: 10.1093/molbev/msq293 21041796 PMC3058770

[149] Mattick JS, Amaral PP, Carninci P, et al. Long non-coding RNAs: definitions, functions, challenges and recommendations. Nat Rev Mol Cell Biol. 2023;24(6):430–447. doi: 10.1038/s41580-022-00566-8 36596869 PMC10213152

[150] Michalak P. Epigenetic, transposon and small RNA determinants of hybrid dysfunctions. Heredity (Edinb). 2009;102(1):45–50. doi: 10.1038/hdy.2008.48 18545265

[151] McDermott SR, Noor MAF. Genetics of hybrid male sterility among strains and species in the *Drosophila pseudoobscura* species group. Evolution. 2011;65:1969–1978. doi: 10.1111/j.1558-5646.2011.01256.x 21729052 PMC3132132

[152] Schaeffer SW, Miller EL. Nucleotide sequence analysis of adh genes estimates the time of geographic isolation of the Bogota population of *Drosophila pseudoobscura*. Proc Natl Acad Sci. 1991;88(14):6097–6101. doi: 10.1073/pnas.88.14.6097 2068088 PMC52029

[153] Prakash S. Origin of reproductive isolation in the absence of apparent genic differentiation in a geographic isolate of *Drosophila pseudoobscura*. Genetics. 1972;72(1):143–155. doi: 10.1093/genetics/72.1.143 5073854 PMC1212808

[154] Ruiz-Garcia M, Alvarez D, Guerrero CJ, et al. Discovery of new rearrangements for the third chromosome in Colombian *Drosophila pseudoobscura* populations and homogeneity of rearrangement frequencies through a whole year. Ann Soc Entomol Fr. 2001;37:393–404.

[155] Kim Y-K, Ruiz-García M, Alvarez D, et al. Sexual isolation between North American and Bogota strains of *Drosophila pseudoobscura*. Behav Genet. 2012;42(3):472–482. doi: 10.1007/s10519-011-9517-7 22065259

[156] Dixon SM, Coyne JA, Noor MAF. The evolution of conspecific sperm precedence in *Drosophila*. Mol Ecol. 2003;12(5):1179–1184. doi: 10.1046/j.1365-294X.2003.01742.x 12694281

[157] Dobzhansky T. Genetic analysis of hybrid sterility within the species *Drosophila pseudoobscura*. Hereditas. 1974;77(1):81–88. doi: 10.1111/j.1601-5223.1974.tb01356.x 4413557

[158] Snook RR. Sperm production and sterility in hybrids between two subspecies of *Drosophila pseudoobscura*. Evolution. 1998;52(1):266–269. doi: 10.2307/2410943 28568153

[159] Orr HA. Genetics of sterility in hybrids between two subspecies of *Drosophila*. Evolution. 1989;43(1):180–189. doi: 10.2307/2409173 28568487

[160] Orr HA, Irving S. Complex Epistasis and the genetic basis of hybrid sterility in the *Drosophila pseudoobscura* Bogota-usa hybridization. Genetics. 2001;158(3):1089–1100. doi: 10.1093/genetics/158.3.1089 11454758 PMC1461699

[161] Gomes S, Civetta A. Hybrid male sterility and genome-wide misexpression of male reproductive proteases. Sci Rep. 2015;5(1):11976. doi: 10.1038/srep11976 26146165 PMC4491705

[162] Gomes S, Civetta A. Misregulation of spermatogenesis genes in *Drosophila* hybrids is lineage-specific and driven by the combined effects of sterility and fast male regulatory divergence. J Evol Biol. 2014;27(9):1775–1783. doi: 10.1111/jeb.12428 24898362

[163] Phadnis N, Orr HA. A single gene causes both male sterility and segregation distortion in *Drosophila* hybrids. Science. 2009;323(5912):376–379. doi: 10.1126/science.1163934 19074311 PMC2628965

[164] Alhazmi D, Fudyk SK, Civetta A. Testes proteases expression and hybrid male sterility between subspecies of *Drosophila pseudoobscura*. G3 Genes GenomesGenet. 2019;9(4):1065–1074. Available from: G3-Genes 10.1534/g3.119.300580 PMC6469408 30723102

[165] Go A, Alhazmi D, Civetta A, et al. Altered expression of cell adhesion genes and hybrid male sterility between subspecies of *Drosophila pseudoobscura*. Genome. 2019;62(10):657–663. doi: 10.1139/gen-2019-0066 31283886

[166] Phadnis N. Genetic architecture of male sterility and segregation distortion in *Drosophila pseudoobscura* Bogota–usa hybrids. Genetics. 2011;189(3):1001–1009. doi: 10.1534/genetics.111.132324 21900263 PMC3213365

[167] Orr HA, Irving S. Segregation distortion in hybrids between the Bogota and USA subspecies of *Drosophila pseudoobscura*. Genetics. 2005;169(2):671–682. doi: 10.1534/genetics.104.033274 15654115 PMC1449097

[168] Noor MAF. Reinforcement and other consequences of sympatry. Heredity (Edinb). 1999;83(5):503–508. doi: 10.1038/sj.hdy.6886320 10620021

[169] Fuller ZL, Haynes GD, Richards S, et al. Genomics of natural populations: how differentially expressed genes shape the evolution of chromosomal inversions in *Drosophila pseudoobscura*. Genetics. 2016;204(1):287–301. doi: 10.1534/genetics.116.191429 27401754 PMC5012393

[170] Kujtan L, Dukas R. Learning magnifies individual variation in heterospecific mating propensity. Anim Behaviour. 2009;78(2):549–554. doi: 10.1016/j.anbehav.2009.05.026

[171] Ortíz-Barrientos D, Noor MAF. Evidence for a one-allele assortative mating locus. Science. 2005;310(5753):1467–1467. doi: 10.1126/science.1121260 16322450

[172] Sturtevant AH, Dobzhansky T. Geographical distribution and cytology of “sex ratio” in *Drosophila pseudoobscura* and related species. Genetics. 1936;21(4):473–490. doi: 10.1093/genetics/21.4.473 17246805 PMC1208687

